# Effect of Charcoal, Probiotic, and Chlorhexidine Mouthwashes on Mechanical Properties and Surface Characterization of Ceramic-Coated Nickel-Titanium Orthodontic Arch Wires: A Comparative In-Vitro Study

**DOI:** 10.7759/cureus.40791

**Published:** 2023-06-22

**Authors:** Peketi Ramya, RSVM Raghu Ram, Inuganti Ranganayakulu, Ghanta Sunil, Bokka Susanthi

**Affiliations:** 1 Orthodontics and Dentofacial Orthopaedics, Ganni Subba Lakshmi (GSL) Dental College and Hospital, Rajahmundry, IND

**Keywords:** ceramic-coated nickel-titanium archwire, surface topography, mechanical properties, chlorhexidine gluconate mouthwash, probiotic mouthwash, charcoal mouthwash

## Abstract

Aim and objectives: To determine the impact on the mechanical properties and surface features of ceramic-coated nickel-titanium (CC-Ni-Ti) archwires when subjected to charcoal, probiotic, and chlorhexidine mouthwashes in in vitro conditions.

Materials and methods: Eighty samples of 25 mm were cut from the posterior end of preformed maxillary 0.016’’ CC-Ni-Ti super elastic archwires (Koden Company, USA) and distributed into four equal groups. Each group of wires was immersed in artificial saliva (Wet Mouth Mouthwash, ICPA Health Products Ltd., India) (control), charcoal mouthwash (Hello activated charcoal extra freshening mouthwash, Hello Products LLC, USA), probiotic mouthwash (Perfora, Probiotic Rinse, India), and 0.2% chlorhexidine gluconate mouthwash (Sensorange, Orange Biotech, Pvt., Ltd., India) (experimental groups) for 90 min at 37 °C. All samples were taken out of their respective solutions and washed with distilled water prior to testing. A three-point bending test was performed on 15 samples from each group using a universal testing device. During the loading and unloading of the archwires, the yield strength (YS), flexural modulus of elasticity (E), and spring back ratio (YS/E) were calculated. The remaining five wires from each group were observed under the scanning electron microscope (SEM) for surface topography evaluation.

Results: The mean differences of loading YS, E, and YS/E between chlorhexidine and charcoal are 302.91 MPa, 4.28 GPa, and 0.0004, whereas unloading values are 172.32 MPa, 4.16 GPa, and 0.0003, respectively, with a statistical significance of <0.001 in terms of YS and E. The mean differences of loading YS, E, and YS/E between charcoal and probiotic are 305.36 MPa, 4.54 GPa, and 0.0005, whereas unloading values are 173.77 MPa, 3.66 GPa, and 0.0003, respectively, with a statistical significance of <0.001 in terms of YS and E. The mean differences of loading YS, E, and YS/E between chlorhexidine and probiotic are 2.45 MPa, 0.26 GPa, and 0.00007, whereas unloading values are 1.44 MPa, 0.49 GPa, and 0.0000533, respectively, with no statistical significance of >0.001 in terms of YS, E, and YS/E. Surface topography alteration was clearly appreciated in the charcoal and probiotic mouthwash groups compared to charcoal mouthwash.

Conclusions: Loading and unloading of 0.016" ceramic-coated nickel-titanium archwires showed an increase in mechanical properties except for the spring back ratio on exposure to chlorhexidine, probiotic, and charcoal mouthwashes. Chlorhexidine and probiotic mouthwashes had a higher yield strength and flexural modulus of elasticity in comparison with charcoal mouthwash and artificial saliva on 0.016" ceramic-coated nickel-titanium archwires. More corrosive changes were seen on 0.016" ceramic-coated nickel-titanium archwires when immersed in chlorhexidine, followed by probiotic and charcoal mouthwashes.

## Introduction

Awareness of aesthetics is increasing day by day, and many people are concerned about their smiles and want well-aligned teeth, which are possible with orthodontic treatment. The concern is not only restricted to correcting the malocclusion but also extends to the appliances used in the treatment. This demand leads to the production of advanced appliances like aesthetic brackets and archwires, which serve both aesthetically and performance-wise in fixed appliance therapy [1]. Fixed orthodontic therapy induces an increase in biofilm formation, leading to a shift in amount, composition, metabolic activity, and pathogenicity of the oral microflora, which in turn has clinical side effects like gingivitis, periodontitis, decalcification of enamel, and dental caries [2]. The aesthetic fixed appliance stays in the oral cavity for a longer duration, similar to that of conventional metal fixed appliances, which might show plaque accumulation and other side effects. Usually, orthodontists recommend their patients use mouthwash along with daily brushing and flossing procedures during orthodontic treatment to prevent tooth decalcification. There are numerous varieties of mouthwashes that are sold commercially; among them, chlorhexidine is the most commonly used one. Chlorhexidine possesses antibacterial properties that are membrane-active, which means that it binds to phospholipids in the inner membrane to increase permeability and let low-molecular-weight substances, such as potassium ions, pass through. Moreover, one significant negative effect of the chlorhexidine molecule's cationic nature is extrinsic tooth discoloration [3]. Due to the disadvantage of staining with long-term usage of chlorohexidine, charcoal mouthwashes and probiotic mouthwashes are gaining popularity. To increase acceptability and help in the fight against halitosis, charcoal and charcoal-containing treatments have been used to clean teeth. These products also contain a variety of inorganic compounds, favourable agents, and botanicals. Historical interest in the use of charcoal-based remedies for intraoral use was ignited by the capacity of coarsely crushed charcoal to abrade away stains and deposits on teeth and absorb large amounts of harmful substances, particularly unpleasant exudates from diseased gums [4]. Improving the hosts' health by altering the microbiome is a hot topic right now. The research has advanced so much that this concept has been introduced into dental mouth rinses. Probiotic mouthwash, for example, includes living bacteria such as Lactobacilli or Bifidobacterium, which lowers the level of Streptococcus mutans in the saliva. Lactobacilli are thought to be part of the normal oral microbiota. They work through a variety of methods, such as competing for the binding site with harmful bacteria, producing antimicrobial chemicals, and modulating the immune response. Probiotic mouthwash was found to be safe and effective for daily use in maintaining dental and periodontal health [5]. In a larger picture, to eliminate dental plaque formation, continuous exposure of different dentifrices and mouthwashes is noted on the fixed appliances, including the aesthetic archwires. Distinct types of attractive archwires available commercially include coated metallic archwires, fibre-reinforced composite archwires, and optiflex archwires. Optiflex archwires lack desirable mechanical characteristics [6]. Fibre-reinforced composite archwires are still in the experimental stage. The only aesthetically pleasing archwires on the market are coated metallic ones. Different materials like Teflon, epoxy resin, and ceramic are used in the coating process [7]. Due to the interaction between the aesthetic archwires and mouthwashes, an alteration in the mechanical and surface characteristics was observed. However, there was not much information available regarding possible mouthwash effects on ceramic-coated nickel-titanium (CC-Ni-Ti) archwires. Therefore, the goal of this in vitro study is to assess how CC-Ni-Ti archwires' mechanical properties and surface features are affected by mouthwashes containing charcoal, probiotics, and chlorhexidine.

## Materials and methods

Methodology

Eighty samples were taken in this investigation based on power analysis and preliminary data, with 80% power and a 95% confidence level for each type of wire. A total of four groups (n = 20 each) meet the constraints of α = 0.05. Of the 20 samples in each group, 15 were for determining the mechanical properties, and the remaining five were for observing the surface topography of the archwires when subjected to various types of mouthwash. Review board clearance from the GSL educational institution, Rajahmundry (Rajamahendravaram), India, was obtained for the use of the in vitro comparative study (GSLEI/IRB/2020/001). Sixty samples of 0.016" preformed maxillary CC-Ni-Ti super elastic archwires (Koden Company, USA) were cut from the posterior part of the straight ends for a length of 25 mm. These wires were divided into four groups at random. Each group was incubated at 37 °C in a separate sterile petri dish with 10 mL of charcoal mouthwash (Hello activated charcoal extra freshening mouthwash, Hello Products LLC, USA), probiotic mouthwash (Perfora, Probiotic rinse, India), 0.2% Chlorhexidine Gluconate mouthwash (Sensorange, Orange Biotech Pvt. Ltd., India), and artificial saliva (Wet mouth mouthwash, ICPA Health Products Ltd., India) (control) for 90 min in an incubator. This amount of time would be similar to three months of daily rinsing with mouthwash for one minute each [8]. Before being put through mechanical testing, samples from the various experimental groups were cleansed with distilled water and transferred to new containers with labels for their respective groups. On a universal testing device (UTES-40-HGFL) with a 5 kN load cell, a three-point bend test was run on 15 samples from each group. The archwire sample is placed on the two poles, which are 12 mm apart, on the stage of the lower jaw of the machine. A steel rod with a bevelled chisel end was positioned in the middle of the archwire section, and a compressive force at a crosshead speed of 0.5 mm/min was applied. Each sample is loaded to a deflection of 3.1 mm and then unloaded to zero deflection at a crosshead speed of 2.5 minutes [8]. Using computer software, each sample's load in Newtons (N) and deflection in millimetres are recorded during both loading and unloading. The engineering beam theory was utilised to calculate the yield strength (YS) and the modulus of elasticity (E) using the data to create load-deflection curves. The spring-back ratio (YS/E) was calculated based on the data obtained [9]. The unloading curves illustrate deactivation forces that give an idea of the wire's possible clinical behaviour, whereas the loading curves produced from the three-point bending test imitate the wire's activation.

Evaluation of surface topography

A scanning electron microscope (SEM) at ×1000 magnification was used to assess the surface characteristics of the archwires. Each sample was put on a holder and examined with a field-emission SEM device (Hitachi, S-3700N). The archwire surface was bombarded with electrons, and each pixel on the SEM image was examined for electron reflection intensity. Based on a visual assessment of the surface irregularities, the surface topographical characteristics were established [8].

The obtained information was computed using IBM SPSS version 20 software (Armonk, NY, USA). The statistical analysis carried out included the means and standard deviations of all the variables. The Kolmogorov-Smirnov test was applied to determine whether the collected data were normal. Both the loading and unloading of the archwire were analysed according to a one-way analysis of variance (ANOVA) with a 5% threshold of significance (=0.05). E, YS, and YS/E for all the tested samples were recorded and compared to see if there were any notable variations between the groups. The data were analysed using Tukey's post hoc testing for multiple pairwise comparisons.

## Results

The distribution of data for all the parameters was analysed using the Kolmogorov-Smirnov test (Table 1). The results showed that the data for YS, E, and YS/E in both the loading and unloading phases followed a normal distribution with p-values ranging from 0.2 to 0.31. These findings suggest that the observed data align reasonably well with the theoretical distributions, indicating the reliability of the measurements and the suitability of the statistical analysis performed.

**Table 1:** Distribution of data with regard to all the study parameters.

Parameter		Kolmogorov-Smirnov statistic	Df	P-value
Loading	Yield strength (MPa)	0.024	60	0.31
	Flexural modulus of elasticity (GPa)	0.068	60	0.2
	Spring back ratio	0.083	60	0.2
Unloading	Yield strength (MPa)	0.0246	60	0.31
	Modulus of elasticity (GPa)	0.065	60	0.2
	Spring back ratio	0.092	60	0.2

The loading and unloading mechanical properties were compared between the control group (artificial saliva) and experimental groups (charcoal mouthwash, probiotic mouthwash, and chlorhexidine mouthwash) (Table 2). For the parameter YS (MPa), the experimental groups showed higher values than the control group during both loading and unloading. Probiotic mouthwash had the highest values, followed by chlorhexidine mouthwash and charcoal mouthwash. In terms of E (GPa), the experimental groups again exhibited higher values compared to the control group for both loading and unloading. Probiotic mouthwash had the highest values, followed by chlorhexidine mouthwash and charcoal mouthwash. Regarding the YS/E, there were no significant differences observed among the mouthwash groups for both loading and unloading. These findings suggest that probiotic and chlorhexidine mouthwashes may have a positive impact on the mechanical properties of dental materials, particularly in terms of YS and E.

**Table 2:** Comparison of loading and unloading mechanical properties between control and experimental groups. One-way analysis of variance.

Parameter		Charcoal mouthwash	Probiotic mouthwash	Chlorhexidine mouthwash	Artificial saliva (control)
Yield strength (MPa)	Loading	872.34 ± 22.09	1177.71 ± 33.36	1175.25 ± 29.63	816.15 ± 20.58
	Unloading	497.22 ± 12.58	671.00 ± 19.01	669.55 ± 16.91	463.94 ± 13.84
Flexural modulus of elasticity (GPa)	Loading	26.77 ± 3.85	31.32 ± 4.97	31.06 ± 2.62	22.54 ± 3.73
	Unloading	23.81 ± 3.87	27.48 ± 4.54	27.97 ± 2.69	19.77 ± 3.65
Spring back ratio	Loading	0.0032 ± 0.0004	0.0037 ± 0.0005	0.0037 ± 0.0004	0.0036 ± 0.0005
	Unloading	0.0020 ± 0.0003	0.0024 ± 0.0003	0.0023 ± 0.0002	0.0024 ± 0.0005

The results of the multiple pairwise comparisons revealed significant differences in mechanical properties between the study groups (Table 3). The probiotic and chlorhexidine mouthwash groups generally exhibited higher values of YS and E compared to the charcoal mouthwash and artificial saliva groups during both the loading and unloading phases. The charcoal mouthwash group had lower values for these parameters. However, there were no significant differences observed in the E between the groups, except for a lower ratio in the charcoal mouthwash group compared to the probiotic mouthwash group during loading.

**Table 3:** Multiple pairwise comparisons of mechanical properties on loading and unloading between the study groups. Tukey’s post hoc tests; *denotes statistical significance.

Parameter	Reference group	Comparison group	Loading		Unloading	
			Mean difference	P-value	Mean difference	P-value
Yield strength (MPa)	Charcoal mouthwash	Probiotic mouthwash	305.36	0.000^*^	173.77	0.000^*^
		Chlorhexidine mouthwash	302.91	0.000^*^	172.32	0.000^*^
		Artificial saliva	56.19	0.000^*^	33.28	.000^*^
	Probiotic mouthwash	Chlorhexidine mouthwash	2.45	0.994	1.44	0.994
		Artificial saliva	361.56	0.000^*^	207.05	0.000^*^
	Chlorhexidine mouthwash	Artificial saliva	359.10	0.000^*^	205.60	0.000^*^
Flexural modulus of elasticity (GPa)	Charcoal mouthwash	Probiotic mouthwash	4.54	0.021^*^	3.66	0.046^*^
		Chlorhexidine mouthwash	4.28	0.012^*^	4.16	0.018^*^
		Artificial saliva	4.23	0.020^*^	4.04	0.023^*^
	Probiotic mouthwash	Chlorhexidine mouthwash	0.26	0.998	0.49	0.984
		Artificial saliva	8.78	0.000^*^	7.70	0.000^*^
	Chlorhexidine mouthwash	Artificial saliva	8.51	0.000^*^	8.20	0.000^*^
Spring back ratio	Charcoal mouthwash	Probiotic mouthwash	0.0005	0.034^*^	0.0003	0.083
		Chlorhexidine mouthwash	0.0004	0.089	0.0003	0.173
		Artificial saliva	0.0003	0.152	0.0003	0.075
	Probiotic mouthwash	Chlorhexidine mouthwash	0.00007	0.978	0.0000533	0.985
		Artificial saliva	0.00012	0.914	0.0000067	1.000
	Chlorhexidine mouthwash	Artificial saliva	0.00004	0.994	0.0000600	0.978

## Discussion

**Figure 1:**
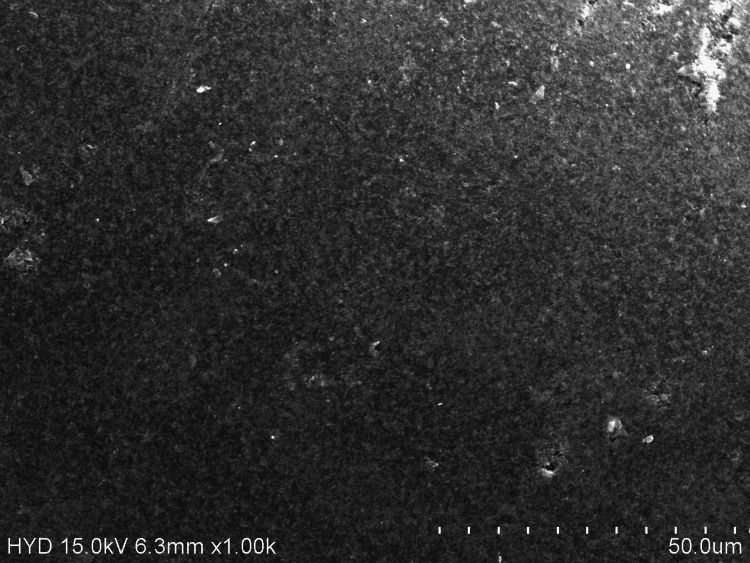
Surface topography of ceramic-coated nickel-titanium archwires exposed to artificial saliva.

Orthodontists commonly prescribe daily topical prophylactic agents to prevent plaque accumulation and demineralization of tooth structure [10]. However, these prophylactic agents get in constant contact with the components of fixed orthodontic appliances like brackets, archwires, etc., which in turn results in the disruption of the titanium oxide layer on nickel-titanium (Ni-Ti) alloys, losing its passivating effect and causing hydrogen embrittlement, leading to an alteration in the mechanical and surface properties of the archwire. These Ni-Ti archwires may have a changed surface as a result of the cosmetic coating, which could reduce the wires' durability, friction, and corrosive qualities [7]. The efficiency of an archwire is significantly influenced by mechanical parameters like YS and E. A low E makes the archwire more flexible, whereas a high YS makes it stiffer or more rigid. These two factors (YS and E) are inversely proportional to each other. By composition and nature, Ni-Ti archwires have a low YS and a high E. These properties get altered when interacting with various solutions, which include mouthwashes that contain chlorhexidine, fluoride, listerine, charcoal, probiotics, etc. The orthodontic alloys' surface has a thin passive oxide coating that prevents corrosion. Disruption of this layer either mechanically or chemically influences the mechanical properties of the archwire. Although coated with ceramic, CC-Ni-Ti archwires, when exposed to mouthwashes and mechanical loads, the outer later gets altered and influences the core archwire material. To investigate the mechanical characteristics and surface characterization of archwires in gustatory research, artificial saliva is frequently used as the preferred control liquid. Artificial saliva mimics human saliva due to the presence of distilled water, sodium bicarbonate, and potassium chloride. Unfortunately, these chloride ions produce a corrosive effect on metal surfaces [11,12]. The findings from the present study show that the YS and E were quite altered upon loading and unloading of the archwire (Tables 2-3). Although the Cu-Ni-Ti archwire is coated with ceramic, when viewed at ×1000 magnification in SEM, a few small black pits were observed (Figure 1).

**Figure 2:**
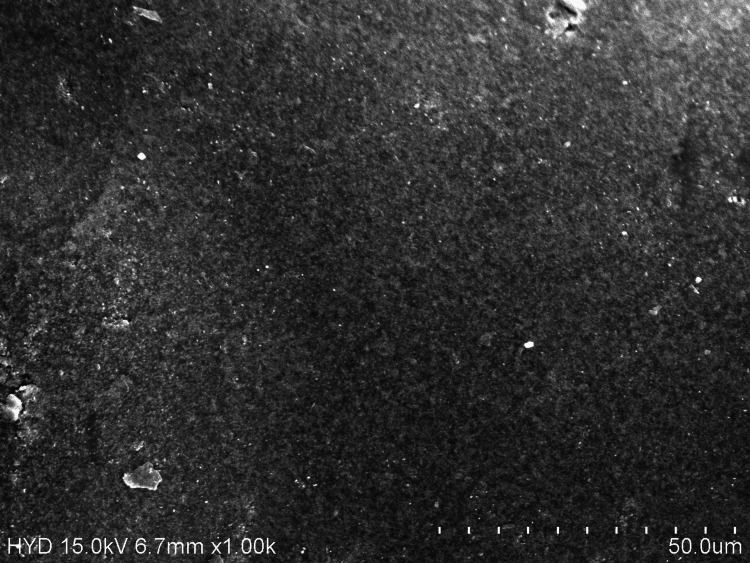
Surface topography of ceramic-coated nickel-titanium archwires exposed to charcoal mouthwash.

The interaction of the components from the artificial saliva on the coated layer or its seepage into the core Ni-Ti would have caused this effect. In a study by Elayyan et al., retrieved 0.016" coated archwires exhibited similar and more severe alterations, including coating delamination across wide areas, discoloration, ditching, and cracking [13]. The values from the present study show that the CC-Ni-Ti archwires subjected to artificial saliva have YS and E close to charcoal mouthwash; however, these were statistically altered when compared to all the groups upon loading and unloading (Tables 2-3). On immersing the archwire into charcoal mouthwash, the surface of the wire showed small black pits under SEM (Figure 2), almost similar to artificial saliva.

**Figure 3:**
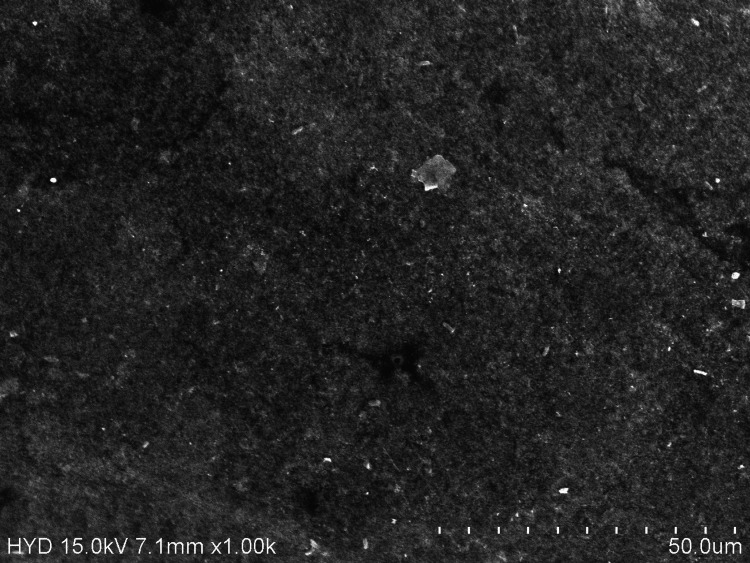
Surface topography of ceramic-coated nickel-titanium archwires exposed to probiotic mouthwash.

Due to their polarity, alcohols, metals like iron and lithium, electrolytes like magnesium, potassium, or sodium, and acids or alkalis cannot be successfully adsorbed by activated charcoal. The abrasiveness of the charcoal particles may be the cause of those pits [14]. Bioremoval of toxic chemicals is usually seen by probiotic microorganisms. These bacteria take up the heavy metal ions using biosorption, entrapment, efflux, reduction, precipitation, and complexation mechanisms [15,16]. Trolic et al. discovered that probiotic bacteria present in the probiotic supplement very slightly increased the likelihood of localised corrosion occurring on metal surfaces. Instead of corrosion being the result of redox processes in which bacteria function as catalysts, this corrosion is more likely to be the result of the deposition of insoluble components of lozenges on orthodontic wires, which causes corrosion underneath the deposit [17]. On immersing the CC-Ni-Ti archwire in probiotic mouthwash, the surface of the wire showed a pitted appearance in addition to deteriorated areas (Figure 3).

**Figure 4:**
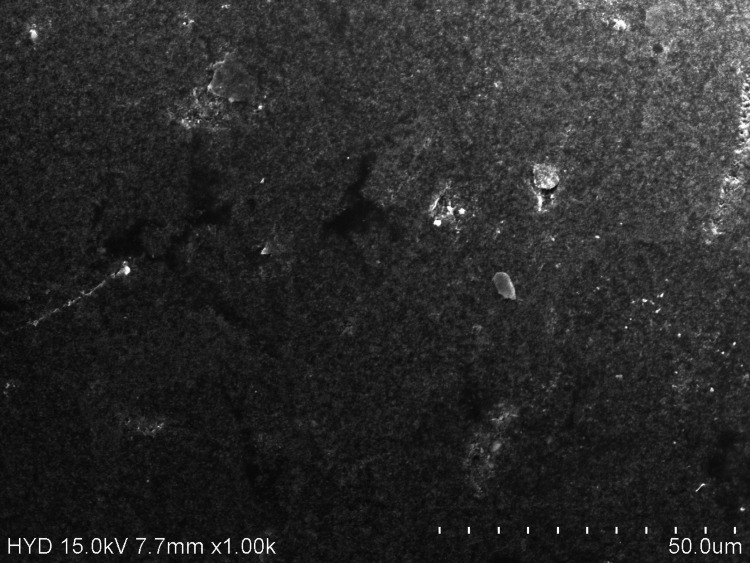
Surface topography of ceramic-coated nickel-titanium archwires exposed to chlorhexidine mouthwash.

The YS and E values in the probiotic mouthwash group are statistically non-significant and very similar to those of the chlorhexidine mouthwash group (Tables 2-3). Because chlorhexidine mouthwash has an acidic pH of 6.5, it corrodes Ni-Ti, thereby releasing large amounts of nickel and chromium ions [18]. In a study by Aghili et al., chlorhexidine mouthwash provided a statistically significant increase in the E of coated wires. In comparison to NiTi wires, coated wires produced lower forces during all loading and unloading intervals with various types of mouthwash, according to their investigation [1]. After immersion in chlorhexidine, mouthwash CC-Ni-Ti archwires in the present study showed numerous black pits and elongated cracking on the outer surface (Figure 4).

The severe change in the surface topography would have let the chlorhexidine solution release the ions and change the YS and E values during the loading and unloading of the archwire (Tables 2-3). Out of all the groups in the present study, artificial saliva showed less pitting and corrosive changes, followed by charcoal, probiotics, and chlorhexidine, respectively, in increasing order. These topography findings are in correlation with the YS, E, and YS/E values when mechanical testing was done under loading and unloading in vitro conditions.

Limitations

This study has certain limitations. First, since it is an in vitro investigation, the findings may not directly translate to clinical performance. The mechanical properties and surface characteristics of CC-Ni-Ti orthodontic archwires can differ in an in vivo setting compared to the in vitro conditions of the study. Second, the study did not consider the influence of oral conditions on the mechanical properties and surface characteristics, which can impact the performance of the archwires in a clinical setting.

Further scope

To validate the findings and assess the effectiveness of charcoal mouthwash, further clinical studies are needed. These studies should consider oral conditions and incorporate patient-specific factors that can influence the mechanical properties and performance of archwires. Conducting comparative studies with a larger sample size and longer follow-up periods would provide more comprehensive insights into the benefits and limitations of using charcoal mouthwash in orthodontic treatments. Additionally, exploring the effects of charcoal mouthwash on other orthodontic appliances and surfaces could expand our understanding of its potential applications.

## Conclusions

The loading and unloading mechanical properties of 0.016" ceramic-coated nickel-titanium archwires dramatically improved after exposure to chlorhexidine, probiotic, and charcoal mouthwashes, with the exception of the spring back ratio. On 0.016" ceramic-coated nickel-titanium archwires, chlorhexidine and probiotic mouthwashes showed greater yield strength and flexural modulus of elasticity than charcoal mouthwash and artificial saliva. All three experimental solutions had an impact on the qualitative surface topography of ceramic-coated nickel-titanium archwires. Greater corrosive changes were seen on 0.016" ceramic-coated nickel-titanium archwires when exposed to chlorhexidine, followed by probiotic and charcoal mouthwashes.
